# Males increase call frequency, not intensity, in response to noise, revealing no Lombard effect in the little torrent frog

**DOI:** 10.1002/ece3.4625

**Published:** 2018-10-31

**Authors:** Longhui Zhao, Xiaoqian Sun, Qinghua Chen, Yue Yang, Jichao Wang, Jianghong Ran, Steven E. Brauth, Yezhong Tang, Jianguo Cui

**Affiliations:** ^1^ Chengdu Institute of Biology Chinese Academy of Sciences Chengdu China; ^2^ Key Laboratory of Bio‐Resource and Eco‐Environment of Ministry of Education, College of Life Sciences Sichuan University Chengdu China; ^3^ University of the Chinese Academy of Sciences Beijing China; ^4^ Ministry of Environmental Protection South China Institute of Environmental Sciences Guangzhou China; ^5^ Ministry of Education Key Laboratory for Tropical Plant and Animal Ecology, College of Life Sciences Hainan Normal University Haikou China; ^6^ Department of Psychology University of Maryland College Park Maryland

**Keywords:** adaptive changes, ambient noise, *Amolops torrentis*, sexual selection, sound communication

## Abstract

Noise is one of the main factors that can influence the processes of sound communication across a wide range of animal groups. Although the effects of ambient noise on animal communication, including anthropogenic noise, have received increasing attention, few studies have examined changes in the fine structure of acoustic signals produced by vocalizing species in constantly noisy environments. Here, we used natural recordings to determine the associations between stream noise and call parameters in the little torrent frog (*Amolops torrentis*). We also used playbacks of stream noise recorded in natural habitats and playbacks of white noise to examine how male vocal signals change with increasing noise levels. The results show that noise intensity has a significant effect on male call frequency, but not on call amplitude or other call characteristics. Based on this evidence, we suggest that in streamside species stream noise drives males to alter call frequency and call as loudly as possible in order to improve discriminability. These findings provide insights into the role played by ecological selection in the evolution of noise‐dependent anuran vocal plasticity.

## 1 INTRODUCTION

Acoustic signals are used by many animal species to find mating partners, integrate spatiotemporal information, deter rivals, and defend against predators (Bradbury & Vehrencamp, 2011). Communication through sound, however, is often constrained by various sources of biotic and abiotic noise (Brumm & Slabbekoorn, 2005; Klump, 1996; Wiley & Richards, 1982). Research on the effects of natural ambient noise and on how animals deal with noise‐related constraints can thus provide insights into the evolution of acoustic communication systems and the potential effect of human activities on these systems (Love & Bee, 2010). Notably, noise and the plastic responses to noise have received increasing interest, particularly with regard to how animals cope with environmental noise (Chan, Giraldo‐Perez, Smith, & Blumstein, 2010; Holt, Veirs, & Veirs, 2008; Nemeth et al., 2013; Parris, Velik‐Lord, & North, 2009).

Several adaptive adjustments are involved in maintaining efficient communication in noise. The acoustic adaptation hypothesis (Morton, 1975) speculates that animals may change vocal frequency in response to ambient noise. The energy of ambient noise is often concentrated in low‐frequency bands, which may overlap with low‐frequency acoustic signals, causing many animals to raise their call frequency for the purpose of increasing the signal‐to‐noise ratio (Goutte, Dubois, & Legendre, 2013; Nemeth & Brumm, 2010). Another familiar strategy is the Lombard effect, in which voice amplitude is increased in response to increasing noise levels (Brumm & Zollinger, 2011). In addition, animals can vary call timing in noisy conditions. For instance, birds and frogs can emit more calls when noise levels are low (Gaston, Warren, & Fuller, 2007).

Noise‐dependent behavioral plasticity often involves multiple changes in signal characteristics, which are often species‐specific and context‐dependent (Hage, Jiang, Berquist, Feng, & Metzner, 2013; Luo, Goerlitz, Brumm, & Wiegrebe, 2015). For instance, studies on echolocating horseshoe bats (*Rhinolophus ferrumequinum*) have shown that shifts in call amplitude and frequency depend on the range of noise the animals are exposed to (Hage et al., 2013; Hage, Jiang, Berquist, Feng, & Metzner, 2014). Nevertheless, we know little about the relationships among these simultaneous adaptive changes and how the relevant mechanisms are linked. While noise‐dependent short‐term vocal plasticity has been well examined, less is known about adaptive changes in constantly natural noisy environments (Brumm & Slabbekoorn, 2005; Röhr, Paterno, Camurugi, Juncá, & Garda, 2015). Yet, these adaptive changes may predict long‐term evolutionary processes associated with fluctuating ambient noise conditions such as increased anthropogenic noise.

Amphibians are excellent study organisms to investigate communication strategies (Starnberger, Preininger, & Hödl, 2014). More specifically, anurans are one of main taxa that rely heavily on acoustic signals for communication, and only a limited number of taxa have been used in previous studies concerning the influence of environmental selection on anuran call characteristics (Boeckle, Preininger, & Hödl, 2009; Penna & Hamilton‐West, 2007; Penna, Pottstock, & Velasquez, 2005). For this reason, more work on the effects of noise on the acoustics of multiple signal characteristics is needed.

The little torrent frog (*Amolops torrentis*) inhabits an environment with persistent high‐level stream noise. During the breeding season, males produce calls consisting of a series of identical repeated notes, from the rocks of mountain streams or near vegetation, throughout the day and night (Zhao, Wang, et al., 2017). Here, we examined the noise‐dependent vocal responses of this species. We investigated the relationships between stream noise and vocal behavior in the natural habitat and determined how males altered vocal characteristics in response to different levels of stream noise and white noise by quantifying the amplitude and spectral–temporal features of calls. Frogs can often change multiple acoustic characteristics in response to increased social density. For this reason, we verified some of our findings by estimating the effect of the density of calling neighbors on vocal behavior.

## 2 MATERIALS AND METHODS

### 2.1 Subjects

All subjects were recorded or tested in streams around the field research bases at the Mt. Diaoluo Nature Reserve (18.44°N and 109.52°E), Hainan Province, China. All experiments were conducted during the day in order to avoid interference with heterospecific calls, although male little torrent frogs can produce acoustic signals and females may lay eggs day and night. Moreover, male calls produced during the day have the same structure as male calls produced at night (L. Zhao, unpublished data). Temperature and humidity are generally known to affect the structural components of frog calls. The general humidity was above 90% and remained relatively stable in the streams. Moreover, experiments were conducted between 0900 hr and 1800 hr and the temperature exhibited only subtle variations (22–25°C) during this period. The humiture was measured using the HOBO Pro V2 (Part No. U23‐001). All tests were carried out stochastically in order to reduce any effect that temperature variation could have on the results.

After the experiments, frogs were toe‐clipped to prevent them from being recorded or tested again. The frogs were then immediately released into the wild. We only marked the leftmost toe of the left leg in order to minimize the potential effect on animal activities. Toe‐clipped subjects did not lose territories and could still emit calls on the same day and night of marking, at the location of capture. All procedures for this study were approved by the management office of the Mt. Diaoluo Nature Reserve and the Animal Care and Use Committee of the Chengdu Institute of Biology, CAS (CIB2016042403). All experiments complied with the present laws of China.

### 2.2 Experimental design

#### 2.2.1 Natural recordings

Sound recordings and measurements followed the procedures described in detail previously (Zhao, Wang, et al., 2017). Briefly, once calling males were located in the stream, the natural calls and running water were recorded immediately using a directional microphone (Sennheiser ME66 with K6 power module) connected to a digital recorder (Marantz PMD 660, 16 bit, 44.1 kHz), while the sound pressure levels of these sounds (SPLs, A‐weighted) were simultaneously measured using a sound level meter (AWA 6291, Hangzhou Aihua Instruments Co.), at a precise distance of 1 m. Since sound radiation varies in its directionality, the microphone and sound level meter were directed along the snout‐vent orientation of the subject. At least six calls were recorded in sequence for each male, and the maximum RMS SPLs (fast time weighting) were also recorded for each call. After each recording session, we immediately measured the level of noise at the position of the frog's head that the male was experiencing. Fifty‐one males were recorded and measured during the breeding season, among which 18 individuals produced calls in the absence of conspecific calling males in the area (isolation) and 33 individuals called in conspecific choruses (aggregation). For the aggregated group, three to five calling males were typically located within 5 m of each other. The degree of acoustic overlapping was relatively low in the little torrent frog choruses, and we measured only focal subject calls and noise that did not overlap the calls of neighbors during the recording sessions.

#### 2.2.2 Noise playbacks

**Figure 1:**
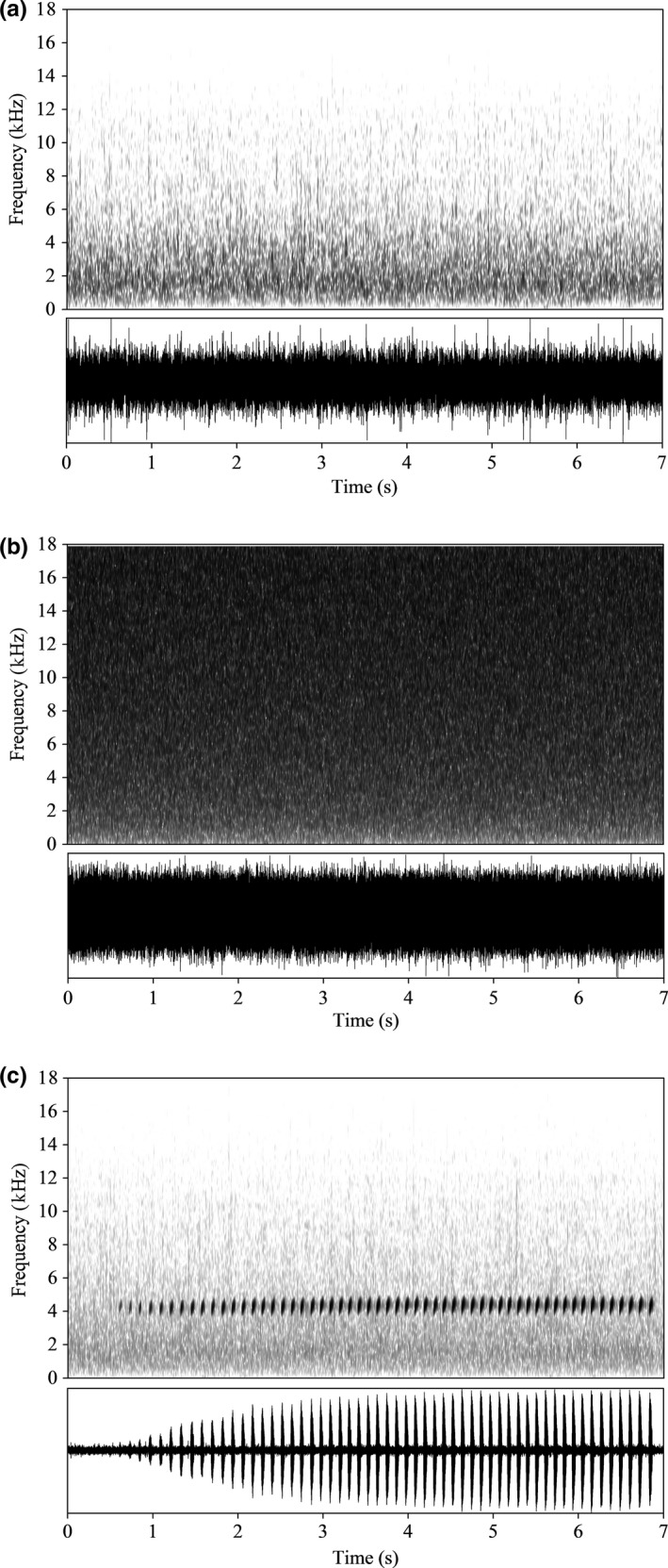
Waveforms (top panel) and spectrograms (bottom panel) of (a) stream noise, (b) white noise, and (c) a typical advertisement call. Stream noise overlaps the signal in the natural environment

Rapidly flowing stream water produces broadband low‐frequency noise with spectral energy decreasing gradually from the low to high frequencies (Figure 1a). The frequency of natural calls ranged from 3.2 to 5.2 kHz (Zhao, Zhu, et al., 2017). Thus, not all signal frequencies would be equally susceptible to masking by stream noise. In view of this, we assessed male vocal responses to the running water and white noise stimuli using the same playback levels (i.e., no noise added, and stream noise and white noise at 10 and 20 dB above background noise). In order to mitigate fluctuations in the amplitude envelope in the noise playback experiments, a standard stream noise stimulus (Figure 1a) was created by combining three stream noise recordings from three typical breeding sites around the field research bases at Mt. Diaoluo. These recordings had approximate amplitudes and were mixed with an operation in Adobe Audition 3.0. White noise (Figure 1b) was generated with Adobe Audition 3.0 software (CA, USA; sampling rate: 44.1 kHz).

Each frog was tested for each of five treatment conditions (i.e., no noise added, and stream noise and white noise at 10 and 20 dB above background noise) in random order with a 1‐min interval between trials. Each treatment lasted 5 min. During playback trials, a sound level meter and directional microphone connected to a digital recorder were used to measure the SPLs of calls and ambient noise (i.e., stream noise plus the playback signal) and to record calls and ambient noise, respectively, at a point 50 cm from the frog aligned with the snout‐vent orientation of the subject. Noise was broadcast from a speaker (JBLCLIP + BLK, JBL) fixed at a position such that the distance between the speaker and the microphone and the speaker and the frog's head was equal, thereby ensuring that the noise intensity was equal at these two points. The speaker was connected to a player. If needed, we adjusted the amplitude of the player noise while the speaker amplitude remained unchanged. In this study, we chose seven males calling at relatively quiet sites, with natural noise intensity levels around 65 dB, as playback subjects. The playback was in addition to the natural background noise from the stream occurring at the site.

#### 2.2.3 Acoustic analyses

**Figure 2:**
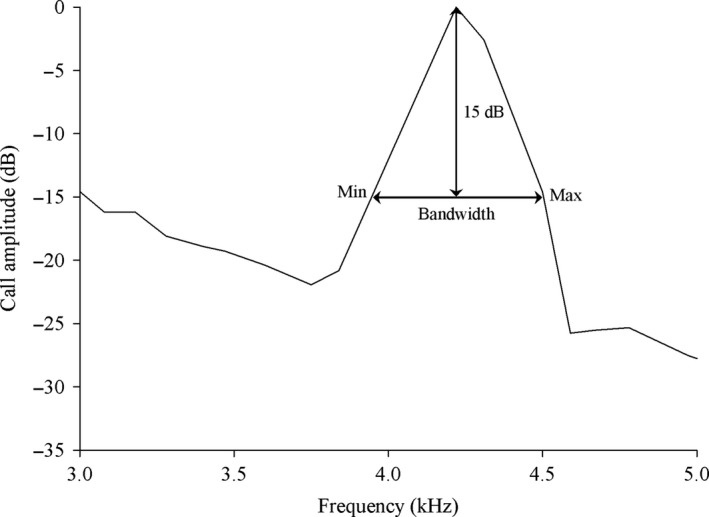
Schematic diagram of the minimum and maximum frequency measurements from power spectra. The minimum and maximum frequency are analyzed at a standard decibel level (here −15 dB) relative to the peak. Bandwidth is the difference between the call minimum and maximum frequency

All frequency measurements were performed with Avisoft‐SASLab Pro (sample rate: 44.1 kHz; FFT size: 1,024; Hamming window). Spectrograms are the representations of energy in frequency bands and are utilized in many experiments requiring sound analyses. Measuring frequencies by eye from spectrograms, however, can produce artifacts that can produce the faulty impression of elevated frequencies in noise (Brumm, Zollinger, Niemelä, & Sprau, 2017; Zollinger, Podos, Nemeth, Goller, & Brumm, 2012), especially for species such as little torrent frogs that call at high‐intensity background noise (Figure 1c). We therefore measured the minimum call frequency, maximum frequency, and bandwidth (the difference between maximum and minimum frequency of call) from power spectra in the present study. For natural recordings, we measured the bandwidth minimum and maximum frequencies at 15 dB below the peak amplitude frequency (Zollinger et al., 2012; Figure 2). In accordance with the work of Zollinger et al. (2012), we chose the measurement threshold based on the lowest signal‐to‐noise ratio (SNR) of the recording. At −15 dB from the peak, we could measure the minimum and maximum frequencies of all recordings above the noise floor. For noise playback recordings, we obtained the same measurements but relative to the peak amplitude across all tests of the same individual. Male calls consist of a series of repeated notes. For each call, we stochastically chose some notes when making the acoustical analysis.

The temporal features of male calls were analyzed using Adobe Audition 3.0 software. For each frog, we determined call duration by averaging across all recorded calls. We determined call rate, defined as the number of calls produced per minute. We determined call effort, defined as the call duration multiplied by the call rate. Signal and noise can overlap in noisy conditions; thus, we subtracted the background noise from the measured amplitude values using logarithmic computation rules as described by Brumm and Zollinger (2011). Stream noise level alone (*L*_noise_) was measured when there were no calling males, and total SPL (*L*_noise+sig_) was measured when focal males emitted complete calls. Then, the SPL of the signal was computed using this formula: *L*_sig_ = 10 log_10_ (10^(^ *^L^* ^noise+sig^ **^/^** ^10)^ – 10^(^ *^L^* ^noise^ **^/^** ^10)^).

### 2.3 Statistical analyses

All statistical analyses were performed with SigmaPlot 11 (Systat Software Inc., San Jose, USA) or SPSS 16.0 (SPSS Inc., USA). The normality of the distributions and the homogeneity of variances were examined using the Shapiro–Wilk and Levene tests, respectively. Pearson correlation analysis was used to determine potential relationships between the level of stream noise and all call properties. A one‐way repeated measures analysis of variance (ANOVA) was carried out to evaluate male vocal responses to stream noise and white noise playbacks, followed by Holm–Sidak post hoc comparisons. A one‐way analysis of covariance (ANCOVA), with noise level as a covariate, was used to evaluate the effect of the density of calling neighbors on vocal behavior. All multiple comparisons were corrected by adjusting *p*‐values using Holm's method. Data were reported as mean ± *SD* (i.e., standard deviation), and *p* < 0.05 was considered significant.

## 3 RESULTS

### 3.1 Relationships between stream noise and vocal behavior

**Figure 3:**
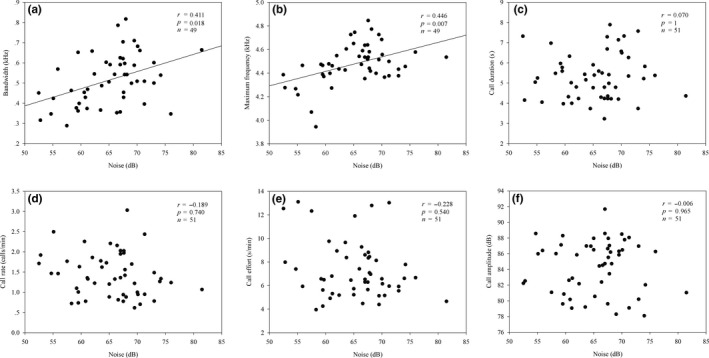
Relationships between stream noise and the (a) call frequency bandwidth, (b) maximum frequency, (c) call duration, (d) call rate, (e) call effort, and (f) call amplitude for little torrent frogs. *p* values were adjusted using Holm's correction for multiple testing

The SPL of ambient noise varied from 52.5 to 81.5 dB during the period we recorded the 51 animal subjects. Theoretically, the density of calling males and overall social environment could affect any of the call traits of interest, but Pearson correlation analysis revealed similar results for the sole and aggregated groups. We therefore determined the relationships between stream noise and call characteristics for all individuals. The results showed that there was no correlation between the minimum call frequency and the SPLs of stream noise (*r* = 0.140, *p* = 1), while the call frequency bandwidth (*r* = 0.411, *p* = 0.018; Figure 3a) and maximum call frequency (*r* = 0.446, *p* = 0.007; Figure 3b) correlated positively with stream noise, across all individuals. Moreover, there were no significant correlations between the SPLs of ambient noise and call duration (*r* = 0.070, *p* = 1; Figure 3c), call rate (*r* = −0.189, *p* = 0.740; Figure 3d), call effort (*r* = −0.228, *p* = 0.540; Figure 3e), and call amplitude (*r* = −0.006, *p* = 0.965; Figure 3f).

### 3.2 The effect of calling neighbor density on vocal behavior

A one‐way ANCOVA revealed that male calling behavior differed between the sole and aggregated groups (Table 1). Males in aggregations produced calls of shorter durations than males that called alone (*p* < 0.001). The sole males, however, produced lower call rates than the aggregated males (Table 1), although this difference did not reach the significance level (*p* > 0.05). As a result, call effort was not significantly different between these groups (*p* > 0.05). Furthermore, mean SPLs also did not differ significantly between the two social environments (*p* > 0.05).

**Table 1:** Mean ± standard deviation for call components for single (*n* = 18) and aggregated (*n* = 33) males experiencing different social pressure levels

Call parameter	Single	Aggregated	*F*_1,50_	*p*	Holm‐*p*
Call duration (s)	6.14 ± 1.04	4.88 ± 0.94	18.9	**<0.001**	**<0.001**
Call rate (calls/min)	1.24 ± 0.52	1.54 ± 0.54	3.68	0.06	0.18
Call effort (sec/min)	7.28 ± 2.40	7.33 ± 2.47	0	0.99	0.99
Call amplitude (dB)	84.0 ± 3.3	84.6 ± 3.4	0.34	0.56	1

The bold values represent the significant *p* values (*p* < 0.05).

### 3.3 Vocal responses to noise playback

**Figure 4:**
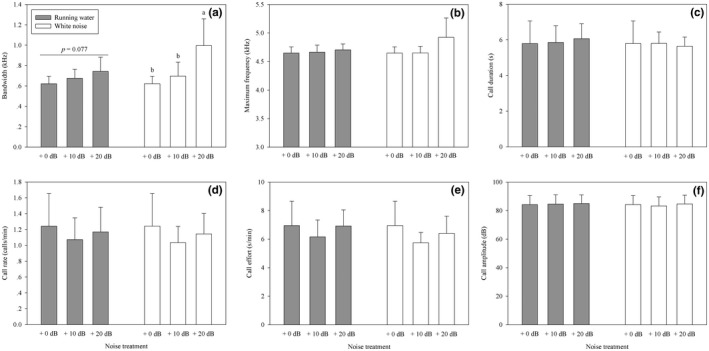
Effects of stream noise and white noise on the (a) call frequency bandwidth, (b) maximum frequency, (c) call duration, (d) call rate, (e) call effort, and (f) call amplitude for little torrent frogs. *p* values were adjusted using Holm's correction for multiple testing. Values which do not share a common superscript letter differ significantly at *p* < 0.05

We found consistent effects for both the running water and white noise treatments by repeated measures ANOVA (Table 2; Figure 4). There was a significant effect of noise intensity on the frequency bandwidth (Figure 4a), but not on the maximum frequency (Figure 4b), minimum frequency (Table 2), call duration (Figure 4c), call rate (Figure 4d), and call effort (Figure 4e). Compared with the control group (no noise added group), the maximum SPLs increased by <1 dB in both stream noise and white noise playback experiments, and the call amplitude did not change significantly with increasing noise levels (Figure 4f).

**Table 2:** Vocal responses to running water and white noise playbacks across various call components (*n* = 7)

Call parameter	Running water			White noise		
	*F*_2,12_	*p*	Holm‐*p*	*F*_2,12_	*p*	Holm‐*p*
Bandwidth (kHz)	6.712	**0.011**	0.077	11.003	**0.002**	**0.014**
Maximum frequency (kHz)	2.529	0.121	0.726	5.301	**0.022**	0.132
Minimum frequency (kHz)	1.483	0.266	1	2.049	0.172	0.516
Call duration (s)	0.653	0.538	1	0.204	0.818	0.818
Call rate (calls/min)	0.641	0.544	0.544	1.195	0.336	0.672
Call effort (sec/min)	1.056	0.378	1	2.074	0.168	0.672
Call amplitude (dB)	0.952	0.413	1	2.233	0.150	0.750

The bold values represent the significant *p* values (*p* < 0.05).

## 4 DISCUSSION

Two mechanisms can be used to explain the fact that many animals raise their call frequency in noise. The acoustic adaptation hypothesis regards this shift as an adaptive adjustment to avoid the low frequencies most susceptible to masking by environmental noise (Morton, 1975). Alternatively, the Lombard hypothesis proposes that the upward shift in frequency may be a consequence of increased call amplitude in noise (Nemeth, Zollinger, & Brumm, 2012; Zhang, Chen, Chen, & Zhao, 2015). In this study, the maximum frequency and bandwidth increased significantly as noise levels increased while the call amplitude did not exhibit any obvious changes. These results support the idea that the adjustment of call frequency is independent of call amplitude in little torrent frogs. Males of this species do not increase call amplitude but call with a higher frequency thereby preventing their acoustic signals from being masked by the background noise.

Some frog species have been reported to call at higher frequencies in noisier environmental conditions (Parris et al., 2009; Vargas‐Salinas & Amezquita, 2013; Zhang et al., 2015). In other species, however, such changes have not been found (Halfwerk, Lea, Guerra, Page, & Ryan, 2016; Jang et al., 2011). Unlike the concave‐eared torrent frog (*Odorrana tormota*) and some birds that increase the minimum frequency resulting in bandwidth narrowing (Francis, Ortega, & Cruz, 2011a, 2011b; Montague, Danek‐Gontard, & Kunc, 2013; Slabbekoorn & Peet, 2003; Zhang et al., 2015), little torrent frogs increase the maximum frequency and bandwidth as noise levels increase. Stream noise energy is mainly concentrated in low‐frequency bands, so the upward shift in the highest frequencies of the calls may be more adaptive than increasing the lowest frequencies in torrent frogs.

Males in some frog species have been described as varying frequency depending on the social context or structure of the chorus. For example, several studies have shown that males can actively alter dominant frequency in particular social situations (Bee & Bowling, 2002; Bee, Perrill, & Owen, 2000; Given, 1999; Wagner, 1989). In this study, we collected a sufficient number of samples (*n* = 51) to determine whether relationships between stream noise and vocal behavior exist, and to identify significant associations between noise intensity and call frequency. Results were also supported by noise playback tests in which the social environment was stable during playbacks. Thus, the apparent shift in frequency is a response to elevated noise levels and not a social response.

Although a plastic increase in call frequency may facilitate sound transmission and detectability in a noisy environment, the potential consequences of such plasticity as a sexually selected trait are not known (Montague et al., 2013). Because high‐frequency signals attenuate more rapidly than low‐frequency signals (Ryan & Kime, 2003), the increase in call frequency would reduce the transmission distance of the acoustic signals, which may reduce signal efficiency for species such as the little torrent frog, that rely heavily on sound communication to attract mates or integrate related information. Furthermore, signal changes in the spectral domain can affect other signaling characteristics. For example, in birds, adjustments of the minimum frequency may decrease song complexity, which can profoundly affect reproductive success (Montague et al., 2013).

The Lombard effect has been widely reported in mammals and birds (Brumm & Zollinger, 2011). A recent study demonstrating the lack of the Lombard effect in a reptile (Brumm & Zollinger, 2017) supports the view that the Lombard effect has evolved independently in birds and mammals, but the condition in amphibians is unresolved. Initially, the existence of a noise‐dependent increase in call amplitude was considered debatable in anurans (Love & Bee, 2010); however, two recent studies have provided important evidence for its occurrence (Halfwerk et al., 2016; Shen & Xu, 2016). In anurans, more studies of noise‐dependent signal amplitude changes are required to elucidate the evolution of the Lombard effect (Halfwerk et al., 2016). For little torrent frogs, stream noise intensity was not correlated with male call amplitude in the natural environment. When males in the present study were exposed to stream noise and white noise, call amplitude did not increase in response to increasing noise levels. These results indicate that male little torrent frogs do not regulate call amplitude as a response to background noise. Thus, the Lombard effect may also evolve independently in anurans in terms of phylogeny.

Call amplitude plays a key role in both sexual selection and signal transmission. For streamside species, high‐intensity stream noise is one of the most important constraints for sound communication (Brumm & Slabbekoorn, 2005). Calling louder can increase the signal‐to‐noise ratio of acoustic signals, increase the number of reproductive opportunities, and hence increase the fitness of male little torrent frogs. Species calling at relatively lower amplitudes might have more flexibility for regulating voice amplitude (Love & Bee, 2010). Accordingly, we suggest that the ubiquitous presence of stream noise may have weakened selection for the Lombard effect.

Recording the responses to noise in a field setting offers some benefits over the methods used by other studies which ran similar tests in the laboratory. However, field experimental methods may not be as accurate as those performed in the laboratory. A previous study suggests that changes in call amplitude in tests of Lombard effect in frogs could be in the range of 1–3 dB (Halfwerk et al., 2016). Our experimental setup may have limitations for identifying differences in amplitude of this magnitude opening the possibility that little torrent frogs can change voice amplitude within a very small range. Such subtle changes, however, would seem to provide little benefit for sound communication in environments of high‐intensity and complex stream noise.

In frogs, changes in call frequency or call amplitude are often accompanied by other changes in vocal components, including call duration, call rate, call effort, and call complexity (Brumm & Slabbekoorn, 2005; Halfwerk et al., 2016; Lengagne, 2008; Shen & Xu, 2016). Nevertheless, similar to the results for call amplitude, other call components including call duration, call rate, and call effort were also not affected by ambient noise in little torrent frogs. Calling by males is energetically very costly (Wells & Taigen, 1986), and vocal production is often constrained by morphological or physiological characteristics. Thus, selection should favor signalers who can adjust signal acoustics in a way that would maximize transmission in environments with variable background noise levels (Love & Bee, 2010). In addition to driving persistently high call amplitude, stream noise would therefore be expected to drive male little torrent frogs to optimize other call components in ways that would improve transmission and discriminability. These ideas are further supported by the analyses of vocal behavior under different social environments. Male call characteristics in anurans, such as the call amplitude and call effort, are often influenced by the calling of other conspecific individuals (Wells & Schwartz, 2007). In the present study, however, higher density of little torrent frog aggregations was not found to be related to higher call effort and call amplitude (Table 1). Temporal parameters such as call rate and call duration influence females choice, and in some species, one parameter could be more important than the others. Call duration was significantly higher in the sole group than in the aggregated group while call rate was not different in the two social environments, suggesting that male little torrent frogs mainly change call duration in response to increased social density.

The amplitude of the environmental noise level experienced by the concave‐eared torrent frog is comparable to that of the little torrent frog (Zhang et al., 2015); however, call amplitude in the concave‐eared torrent frog can increase from 70–75 to 80–85 dB when exposed to 53–83 dB noise (Shen & Xu, 2016). In a previous study, ambient noise and call characteristics were compared across three streamside species (Zhao, Wang, et al., 2017). The results showed that the little torrent frog had lower dominant frequency (*A. torrentis*: 4,318 Hz; *Micrixalus saxicola*: 4,771 Hz; *Staurois parvus*: 5,578 Hz) and higher call amplitude (signal/noise: *A. torrentis*: 80.3/62.4; *M. saxicola*: 69/67; *S. parvus*: 62/72) when compared to the other two species. Similar to the concave‐eared torrent frog, these two torrent species can produce natural calls with relatively higher frequency and lower amplitude and therefore would seem to possess more flexibility for regulating voice amplitude. Future work is needed to determine whether these species can adjust vocal amplitude when exposed to increased noise levels.

In summary, little torrent frog males exhibit significant frequency plasticity in response to increased ambient noise; however, male call amplitude and other call components do not change with increased noise levels. Thus, we find no Lombard effect in little torrent frogs. We suggest that stream noise drives male little torrent frogs to alter call acoustics in a way that would theoretically improve discriminability while continuing to maximize call amplitude. More studies of other streamside breeding species that produce multicomponent calls will provide valuable insights into the evolution of plasticity mechanisms that are adaptive in noisy environments.

## CONFLICT OF INTEREST

We have no competing interests.

## AUTHOR CONTRIBUTIONS

LZ and JC conceived and designed the research. LZ, JW, YY, and XS performed the experiments. LZ, JC, SEB, YT, QC, and JR analyzed the data and wrote the paper. All authors read and approved the final version.

## DATA ACCESSIBILITY

Data used to generate the results and figures are available from the Dryad Digital Repository: https://doi.org/10.5061/dryad.54fp3j1.
